# A retrospective analysis of commonly prescribed medications and the risk of developing breast cancer related lymphedema

**DOI:** 10.15761/crt.1000293

**Published:** 2020-02-28

**Authors:** Eelco FJ Meijer, Echoe M Bouta, Clive Mendonca, Melissa N Skolny, Laura W Salama, Alphonse G Taghian, Timothy P Padera

**Affiliations:** 1 Edwin L. Steele Laboratories for Tumor Biology, Massachusetts General Hospital, Boston, Massachusetts 02114, USA; 2 Department of Radiation Oncology, Massachusetts General Hospital Cancer Center, Boston, Massachusetts 02114, USA; 3 Harvard Medical School, Boston, Massachusetts 02115, USA; 4 Trinity Life Sciences, Waltham, Massachusetts 02451, USA

**Keywords:** BCRL, breast cancer, lymphedema, severity, treatment

## Abstract

**Objectives:**

Breast cancer related lymphedema (BCRL) is a common complication of current breast cancer treatment modalities, significantly lowering quality of life for these patients and often leading to recurrent infections. Here, based on pre-clinical literature, we aim to retrospectively evaluate the risks of prescribed medications on BCRL development.

**Methods:**

All post-operative breast cancer patients who received radiotherapy from 2005–2013 at Massachusetts General Hospital and developed lymphedema(n=115) were included in the analysis. Comparable patients without lymphedema(n=230) were randomly selected as control. The following classes of medications were analyzed: NSAIDs, corticosteroids, angiotensin system inhibitors, calcium channel blockers and hormonal therapy. Known risk factors for lymphedema development were included as variables, including BMI, age at diagnosis, type of surgery, number of lymph nodes removed and radiation therapy. Outcomes were BCRL development and lymphedema severity.

****Results**:**

Similarly, to previous studies, we found that an increase in BMI increases the risk of BCRL(p=0.006) and axillary lymph node dissection has a higher risk of developing BCRL compared to sentinel lymph node biopsy(p=0.045). None of the drugs studied increased the risk of BCRL development or lymphedema severity. However, lymphedema severity was positively correlated with the number of lymph nodes removed(p=0.034).

****Conclusion**:**

We found that anti-inflammatory drugs, anti-hypertensive drugs and hormonal therapy taken during the year postoperatively do not increase the risk of BCRL development or lymphedema severity in breast cancer patients. While others have demonstrated that the number of lymph nodes removed during surgery increases the risk of BCRL, we found it also correlates to lymphedema severity.

## Introduction

Over a million women are diagnosed annually with breast cancer worldwide, accounting for approximately a quarter of all diagnosed cancers in women [1,2]. These women subsequently undergo treatment that can entail a combination of surgical intervention, radiation therapy, chemotherapy, hormonal therapy and/or targeted therapy. The five year survival rate for stages of breast cancer from carcinoma *in situ* thru invasive cancer, is now around 90% [1]. For these patients, lymphedema of the upper extremity is one of the most well-known long-term complications of surgical intervention, including axillary lymph node dissection (ALND) and/or sentinel lymph node biopsy (SLNB) [3]. Depending on the postoperative treatments, the incidence of breast cancer related lymphedema (BCRL) has been reported to range between 2% and 56%, significantly lowering quality of life and increasing the risk of skin and soft tissue infections (SSTIs) in these patients [2,4].

Lymphedema consists of interstitial accumulation of protein-rich fluid combined with inflammation, adipose tissue hypertrophy and progressive fibrosis [5]. Lymphedema can lead to functional impairment, physical deformity and SSTIs of the affected limbs. It is estimated that there are 10 million patients in the United States currently afflicted with lymphedema [6–8]. Of these it is estimated that over seven million meet the criteria for BCRL. Known major risk factors for BCRL include high body mass index (BMI), radiation therapy, a greater number of lymph nodes removed during surgery, the location of removed lymph nodes and high blood pressure [9]. Current treatments may ameliorate symptoms in these patients, but there is no curative therapy known. Identifying preventative measures and therapeutic options for these patients will improve quality of life for millions of patients worldwide.

Adjuvant radiation therapy following lumpectomy has been shown to reduce the risk of in-breast recurrence and metastasis [10–12] and overall survival [13]. However, radiation therapy has been repeatedly confirmed as a risk factor in the development of BCRL [14,15]. In the study from Warren, *et al.* [11], the lymphedema risk in patients, defined as 10% arm volume difference, increased from 3–7% to 21–24% with the addition of regional lymph node radiation. Unfortunately, little is known about the mechanisms responsible for this effect. Previous studies have shown that VEGF-C sensitizes lymphatic endothelial cells to a state of radiation induced permanent senescence [16], potentially limiting reparative lymphangiogenesis. Further, radiation therapy is known to cause tissue fibrosis—a hallmark of lymphedema—as a result of transforming growth factor (TGF)-β-dependent mechanisms [17,18]. Fibrosis is also a critical inhibitor of lymphatic regeneration [19] and TGF-β has been shown to inhibit lymphatic vessel formation [7,20,21].

The effects of angiotensin receptor blockers (ARBs) on TGF-β driven fibrosis have been studied in various pathologies [22–25], demonstrating promising results in reducing fibrosis in many different tissues. Angiotensin converting enzyme (ACE) inhibitors have been shown to have similar, but lesser, anti-fibrotic effects [26]. Even though previous studies have shown that fibrosis reduces the functional regeneration of lymphatics, a literature search for the effects of ARBs or ACE inhibitors (together angiotensin system inhibitors, ASIs) on lymphedema and/or fibrosis after breast cancer treatment in humans yielded no results, motivating this retrospective study.

Other therapies could also hypothetically affect the development of BCRL. Inflammation is a hallmark of lymphedema, and non-steroidal anti-inflammatory drugs (NSAIDs), including aspirin, exert their anti-inflammatory effects through COX-2 inhibition [27]. Pre-clinically, COX-2 specific inhibition has been shown to restore lymphatic contractility depressed by the inflammatory cytokine IL-1β in rats [28]. In addition, various other circulating inflammatory mediators are known to modulate lymphatic function [29]. Medication prescribed for hormone positive disease in breast cancer patients and steroids have also shown anti-inflammatory and anti-fibrotic effects [30–32]. Moreover, some forms of primary lymphedema are known to develop at or shortly after the onset of puberty [5], suggesting possible hormonal influences on developing lymphedema in these patients. Calcium channel blockers (CCBs), used to treat hypertension in patients, have also been shown in animal studies to inhibit lymphatic function [33].

Based on potential drug interactions with lymphatic vessel function defined by preclinical literature, our aim was to retrospectively evaluate the effects of prescribed medication on BCRL development in 115 patients who underwent breast cancer surgery in the years 2005–2013 and received postoperative follow- up at Massachusetts General Hospital. Finding a differential risk for BCRL associated with prescribed medications could have a major clinical impact by reducing morbidity in millions of patients worldwide and reducing healthcare costs in battling this dreaded, yet common, complication of current breast cancer treatment modalities. Below we describe our retrospective analysis on the above-mentioned medication classes and their potential effect on developing BCRL.

## Methods

### Patient Population

After the approval from the Massachusetts General Hospital’s Institutional Review Board, anonymous data were retrospectively collected from medical records of breast cancer patients that underwent surgery between 2005–2013 at our institution. The patients in our study were closely monitored for lymphedema and participated in a screening program [34] with follow-up until 2015. From the 811 breast cancer patients without lymphedema, 230 were randomly selected to be included. All patients with lymphedema (n=115), defined as having a relative volume change (RVC) ≥ 10% of the arm, were included [35]. Data retrieved from the medical records were BCRL development, severity of lymphedema measured by RVC, and known risk factors for BCRL development: BMI, type of surgery, number of lymph nodes removed and radiation therapy (Table 1). RVC was calculated using perometry, a volume measurement technique utilizing an array of moving optoelectronic infrared sensors. Every patient had their arm measured pre-operatively (baseline arm measurement) and postoperatively, concurrently with chemotherapy infusions or radiation therapy and then at 3–7 months intervals following treatment. RVC reported here is the average of the last 6 months of follow up in the BCRL patients. BMI was measured pre-operatively.

In addition to the known risk factors of BCRL, drug usage of the following was recorded: NSAIDs, corticosteroids, aspirin, ASIs, CCBs and hormonal therapy (Table 2). Only medications initiated before surgery and taken for at least 1 year postoperatively were included. Hormonal therapy was generally initiated within 4 months after surgery and was also included. If patients switched hormonal therapy within a year postoperatively, we included the longest used drug, which was always the second drug in this cohort. We did not look at the effects of drugs initiated after lymphedema diagnosis.

### Statistical Analysis

Data was analyzed using IBM SPSS Statistics v22.0. Univariate analysis was performed using a chisquare or Fisher’s exact test for all categorical variables, or a two-tailed t-test. Multivariable logistic regression was used to assess the risk factors for developing BCRL. Multiple regression was used to determine if the RVC, a quantified metric for severity of BCRL, can be predicted by any of the hypothesized risk factors.

## Results

### Patient Population

Out of the 345 patients in this study, the average BMI from 342 patients was 28.0 ± 6.0 kg/m^2^ (mean ± standard deviation) and age at breast cancer diagnosis was 56.9 ± 11.8 (Table 1). Among the total patient population, 199 patients (57.7%) received SLNB versus 109 (31.6%) undergoing ALND. The other 37 patients (10.7%) did not undergo SLNB or ALND. The average number of lymph nodes removed during ALND was 6.6 ± 8.1. The majority of patients (80.9%) received radiation therapy, with 183 (53%) receiving partial or total breast irradiation and 96 (27.8%) receiving regional lymph node radiation (RLNR). In patients with BCRL, the mean RVC was 10.5 ± 9.0.

### Univariate Analysis

Univariate analysis revealed a significant difference in the frequency of BCRL based on patient BMI at the time of surgery, axillary surgery performed, the number of lymph nodes removed, the use of radiation therapy, neoadjuvant and adjuvant chemotherapy treatment, and the use of ASIs (Table 3). BCRL patients had a significantly higher BMI and a greater number of lymph nodes removed than patients without lymphedema (p<0.001). A significantly higher rate of ALND and lower rate of SLNB procedures were noted in BCRL patients compared to patients without lymphedema (p<0.001). There was a greater proportion of patients taking ASIs in the BCRL group (24.3%) when compared to those who did not develop BCRL (15.2%).

### Multivariable Analysis

**Figure 1.**
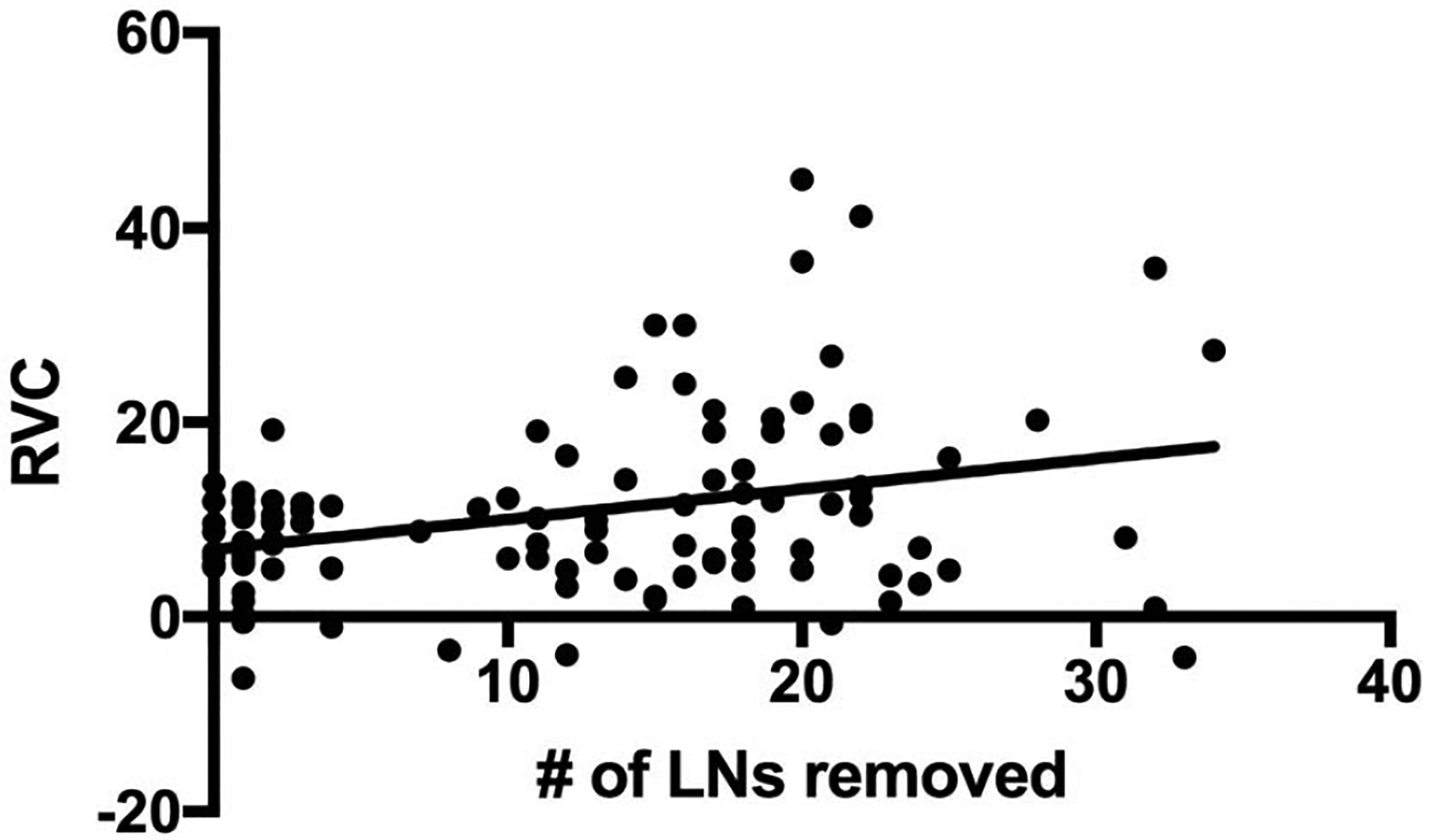
Linear correlation between RVC and number of lymph nodes (LNs) removed Multiple regression was performed with the arm RVC outcome and the potential risk factors from Table 1. Only the number of LNs removed was correlated to RVC and is plotted here as a univariate.

Our results show that an increase in BMI increases the risk of BCRL (Table 4). In addition, our results show that SLNB has a 72% lower risk of developing BCRL versus ALND. Due to sample size limitations, a selection of variables was included for analysis. None of the drugs studied were statistically significant in our logistic regression analysis, leading us to conclude that use of the selected drugs does not affect the formation BCRL. Using additional multiple regression analysis, the only significant predictor of RVC was the number of lymph nodes removed (coefficient=0.36, p=0.034, plotted as a univariate in Figure 1).

## Discussion

While univariate analysis showed that several risk factors were statistically related to the frequency of BCRL, only BMI and the axillary surgery were found statistically significant in the multivariable analysis. These data further confirm that ALND [36–38] and BMI [39–42] are risk factors for developing BCRL (Table 4). In addition, we found that several parameters considered known risk factors for BCRL, such as regional radiation therapy and number of lymph nodes removed, were not statistically significant in our sample population (Table 4).

In our cohort, univariate analysis showed a significantly higher proportion of patients with lymphedema received radiotherapy or RLNR, which targeted the supraclavicular and axillary regions. There was a greater proportion of patients taking ASIs in the BCRL group (24.3%) when compared to those who did not develop BCRL (15.2%), contrary to our hypothesis that ASIs would reduce risk of BCRL by inhibiting the formation of post-treatment fibrosis. As ASI treatment was only significant in the univariate analysis, this might suggest that ASI use could be related to BMI. In general, there may be an increase in prevalence of treatment resistant hypertension in obese patients, which could result in a greater likelihood of a prescription for ASIs. A power analysis (data not shown; power=80%, alpha=0.05) revealed that in order to detect a difference between ASI use and BRCL outcomes on multivariable analysis, we would need larger sample totaling 900–1000 patients given the current sample’s probability of ASI use and incidence of BRCL.

The patients in our study were closely monitored for lymphedema and participated in a screening program [34]. This program is patient specific and some received aggressive treatment, including the use of compression garments, range-of-motion exercises, massage, intensive bandaging and, in select cases, additional surgery. While others have demonstrated that the number of lymph nodes removed increases the risk of lymphedema [43], our data show that the number of lymph nodes removed correlates to the severity (Figure 1). In this context, it is important for patients with many lymph nodes removed to participate in lymphedema screening programs to promote early intervention for BCRL.

In this study, we looked at hormone therapy and other groups of medications initiated before surgery and taken for at least one year postoperatively. Other medications, disease characteristics, neoadjuvant care, as well as specific prescriptions or comorbidities could not be included in the analysis due to the relatively low patient sample size. Additionally, postoperative follow-up in these patients ranged from 2–10 years, while average time until lymphedema development in our cohort was approximately 15 months (Table 1). In addition to patients not experiencing disease progression at the same rate, patients are inherently in different stages of disease when analyzing RVC.

The challenge of looking retrospectively at the effect of drugs on the development and severity of lymphedema is the extensive comorbidities that are associated with the reason why the medications were prescribed and the effect of these comorbidities on lymphatic function. While we hypothesized ASIs would reduce the risk of BCRL, our data trended toward the opposite effect on univariate analysis. We are unable to conclude if this is due to the possibility that hypertension predisposes patients to BCRL [44], which could hypothetically outweigh any positive ASI effects in hypertensive patients. In normotensive patients, therapies with anti-fibrotic effects, such as ASIs, might be beneficial. Furthermore, ASIs may have other effects on BCRL that we did not hypothesize and may be prescribed for other medical indications than hypertension. NSAIDs are commonly prescribed for chronic inflammatory conditions. Inflammation can impair lymphatic function through production of cytokines that inhibit lymphatic pumping [28,29,45]. Thus, if patients with chronic inflammation are at a greater potential risk of BCRL, the use of NSAIDs might normalize this risk back to that of the general population. Our retrospective study would not be able to detect this risk reduction. Further, over the counter drug purchases might not be recorded in the medical records at all, making our dataset incomplete for NSAID use.

CCBs do not seem to have any negative effect on developing BCRL or the severity of lymphedema in our study, even though specific CCBs have been shown to reduce lymphatic function in animal experiments [33]. This indicates that there might be no contraindication for prescribing CCBs in patients at risk for developing BCRL.

## Conclusions

This study represents a first attempt to observe if commonly prescribed medications can affect the risk of developing BCRL. We determined that in our sample neither anti-inflammatory, anti-hypertensive or hormone therapies alter the risk of developing BCRL, which all have been shown to effect lymphatic function or tissue fibrosis pre-clinically. In addition, we found that the number of lymph nodes removed correlates not only to the risk of BRCL, but also to the severity. It should be noted that for the medications, patient numbers were small, leading to an underpowered analysis for small effect sizes. Thus, further investigation with a larger cohort is warranted for these drugs.

## Figures and Tables

**Table 1.** Clinical characteristics of patient cohort

	Mean (range)	n (%)
**BCRL**		115
**RVC in BCRL (N=115)**	10.5 (−6.25–45.02)	
**BMI**	28.0 (17.04–55.67)	
**Age at BC diagnosis**	56.9 (24–86)	
**Months to BCRL development**	15.3 (1.71–83.87)	
**Surgical technique**		
Lumpectomy		236 (68.4%)
Mastectomy		109 (31.6%)
**Axillary surgery**		
None		37 (10.7%)
SLNB		199 (57.7%)
ALND		109 (31.6%)
**Number of LNs removed**	6.6 (0–34)	
**Radiation therapy**		
None		66 (19.2%)
Partial or total breast irradiation		183 (53.0%)
Total breast + subclavicular and/or axillary irradiation (RLNR)		96 (27.8%)
**Adjuvant chemotherapy**		138 (40.0 %)
**Neoadjuvant chemotherapy**		40 (11.6%)
**NSAIDs**		33 (9.6%)
**Calcium channel blockers**		20 (5.8%)
**Steroids**		2 (0.6%)
**Aspirin**		59 (17.1%)
**Angiotensin system inhibitors**		63 (18.3%)
**Hormone therapy**		
None		97 (28.2%)
SERMs		116 (33.7%)
Aromatase inhibitors		131 (38.1%)

BCRL: breast cancer related lymphedema; RVC: relative volume change; BMI: body mass index; LNs: lymph nodes; NSAIDs: Nonsteroidal anti-inflammatory drugs; SERMs: selective estrogen receptor modulators.

**Table 2.** List of drugs in each category ASI: Angiotensin system inhibitor; CCB: Calcium channel blocker; NSAID: Non-steroidal anti-inflammatory drug.

ASIs	CCBs	Corticosteroids	Hormonal therapy	NSAIDs
Valsartan	Amlodipine	Prednisone	Anastrozole	Aspirin
Losartan	Nifedipine	Dexamethasone	Tamoxifen	Ibuprofen
Irbesartan	Felodipine		Letrozole	Naproxen
Olmesartan	Diltiazem		Exemestane	Celecoxib
Azilsartan			Toremifene	Meloxicam
			Raloxifene	Sulindac

**Table 3.** Univariate analysis for BCRL risk factors

	Mean (SD) or n (%)		
	BCRL	No BCRL	Overall p-value
**BCRL**	115 (33.3%)	230 (66.7%)	-
**BMI**	29.8 (5.89)	27.1 (5.68)	**< 0.001**^**&**^
**Age at BC diagnosis**	57.89 (11.31)	56.38 (11.98)	0.254^&^
**Surgical technique**			
Lumpectomy	73 (63.5%)	163 (70.9%)	0.164^
Mastectomy	42 (36.5%)	67 (29.1%)	
**Axillary surgery**			
None	8 (7.0%)	29 (12.6%)	
SLNB	36 (31.3%)	163 (70.9%)*	**< 0.001**^
ALND	71 (61.7%)	38 (16.5%)*	
**Number of LNs removed**	11.57 (9.56)	4.41 (5.83)	**< 0.001**^**&**^
**Radiation therapy**			
None	13 (11.3%)	53 (23.0%)*	
Partial or total breast irradiation	41 (35.7%)	142 (61.7%)*	**< 0.001**^
Total breast + subclavicular and/or axillary irradiation (RLNR)	61 (53.0%)	35 (15.2%)*	
**Adjuvant chemotherapy**	61 (53%)	77 (33.5%)	**.0004**^**&**^
**Neoadjuvant chemotherapy**	20 (17.4%)	20 (8.7%)	**.017**^**&**^
**NSAIDs**	11 (9.6%)	22 (9.6%)	1.000^&^
**Calcium channel blockers**	7 (6.1%)	13 (5.7%)	0.871^&^
**Steroids**	2 (1.7%)	0 (0%)	0.11^#^
**Aspirin**	24 (20.9%)	35 (15.2%)	0.189^&^
**Angiotensin System inhibitors**	28 (24.3%)	35 (15.2%)	**0.039**^**&**^
**Hormone therapy**			
None	25 (21.9%)	72 (31.3%)	
SERMs	36 (31.6%)	80 (34.8%)	0.056^
Aromatase inhibitors	53 (46.5%)	78 (33.9%)*	

Significant (p<0.05) differences using univariate analysis in the frequency of BCRL based on patient BMI at the time of surgery, axillary surgery performed, the number of lymph nodes removed, the use of radiation therapy, neoadjuvant and adjuvant chemotherapy treatment, and the use of ASIs.

Overall p-value for category from a ĉhi-square, ^#^fisher’s exact test, or ^&^two-tailed t-test; *frequencies significant (p<0.05) by a z-test. BCRL: breast cancer related lymphedema; BMI: body mass index; SLNB: Sentinel lymph node biopsy; ALND: Axillary lymph node dissection; LNs: lymph nodes; RLNR: Regional lymph node radiation; NSAIDs: Nonsteroidal anti-inflammatory drugs; SERMs: selective estrogen receptor modulators.

**Table 4.** Multivariable analysis for BCRL risk factors Multivariable analysis using the full information logistic regression model. Due to sample size limitations, a selection of variables was included for analysis. BCRL: breast cancer related lymphedema; BMI: body mass index; SLNB: Sentinel lymph node biopsy; ALND: Axillary lymph node dissection; LNs: lymph nodes; RLNR: Regional lymph node radiation; NSAIDs: Nonsteroidal anti-inflammatory drugs; SERMs: selective estrogen receptor modulators.

	Coefficient	Standard Error	p	Odds Ratio
**BMI**	0.065	0.024	0.006*	1.067 (1.019–1.118)
**Age at BC Diagnosis**	0.024	0.016	0.133	1.024 (1.019–1.118)
**Axillary Surgery**			0.067	
None vs. SLNB	−0.704	0.801	0.379	0.495 (0.103–2.376)
SLNB vs. ALND	−1.268	0.634	0.045*	0.281 (0.081–0.974)
**Number of LNs removed**	0.038	0.036	0.295	1.038 (0.968–1.114)
**Radiation therapy**			0.171	
None vs. Total breast + subclavicular and/or axilla irradiation	−0.961	0.515	0.062	0.383 (0.139–1.050)
Partial or total breast irradiation vs. Total breast + subclavicular and/or axilla irradiation (RLNR)	−0.571	0.426	0.179	0.565 (0.245–1.301)
**NSAIDs**	0.017	0.471	0.971	1.017 (0.404–2.559)
**Calcium channel blockers**	−1.078	0.634	0.089	0.340 (0.098–1.178)
**Steroids**	20.365	3×10^4^	0.999	7×10^8^ --
**Aspirin**	0.291	0.385	0.449	1.338 (0.629–2.846)
**Angiotensin system inhibitors**	0.467	0.377	0.216	1.596 (0.762–3.343)
**Hormone therapy**			0.173	
None vs. Aromatase inhibitors	−0.460	0.367	0.209	0.631 (0.308–1.294)
SERMs vs. Aromatase inhibitors	0.252	0.392	0.520	1.287 (0.597–2.773)
**Constant**	−3.057	1.400	0.029	0.047

## Abbreviations:

ACE: Angiotensin converting enzyme
ALND: Axillary lymph node dissection
ARB: Angiotensin receptor blocker
ASI: Angiotensin system inhibitor
BCRL: Breast cancer related lymphedema
BMI: Body mass index
CCB: Calcium channel blocker
NSAID: Non-steroidal antiinflammatory drug
RLNR: Regional lymph node radiation
RVC: Relative volume change
SERM: Selective estrogen receptor modulator
SLNB: Sentinel lymph node biopsy
TGF: Transforming growth factor
BC: Breast cancer
